# Obituary

**Published:** 1887-01

**Authors:** 


### Obituary.

Died December 1st, in Milwaukee, at his residence, Dr. J. S. Perkins, dentist. His death was the result of his own act, the discharge of a pistol shot through his head. He died in a few moments after the discharge of the weapon. He was the son of Dr. D. W. Perkins, who for many years was a prominent dentist and citizen of Milwaukee. The deceased was thirty-seven years of age; he had been in active practice since 1878. Of Dr. Perkins an intimate friend says: "He had an even disposition, and seemed inclined to take things in a matter of fact way. I do not believe he had an enemy in the world. He was liberal to a fault, and was neither a drinking nor a dissipated man. I have known him for years, and never noticed any thing strange in his actions. He had been greatly afflicted for some time past with neuralgia, and had taken a good deal of morphine as a remedy. He was in a state of mind, induced undoubtedly, by the disease and morphine in which the distructive act was committed. No one had even heard an intimation of such a thing from him. He leaves a widow and two children. He was quite active in professional matters in his own State, was a member of the State Dental Society, and was one of the organizers of the Odontological Society of Milwaukee.

